# Regulation of p53 oligomerization by Ras superfamily protein RBEL1A

**DOI:** 10.18632/genesandcancer.71

**Published:** 2015-07

**Authors:** Ki Lui, M. Saeed Sheikh, Ying Huang

**Affiliations:** ^1^ Department of Pharmacology, State University of New York, Upstate Medical University, Syracuse, NY, USA; ^2^ Division of Science and Technology, The Hong Kong Polytechnic University, Hong Kong Community College, Hung Hom, Kowloon, Hong Kong

**Keywords:** RBEL1A, Ras superfamily GTPase, p53 tumor suppressor, oligomerization

## Abstract

Our previous studies showed that RBEL1A overexpressed in multiple human malignancies and its depletion by RNAi caused severe growth inhibition in tumor cells. We also showed that RBEL1A directly interacted with p53 and such interactions occurred at the oligomeric domain of p53. However, the effect of such interactions on p53 oligomerization and function remained to be investigated. Here, we report that the interaction of RBEL1A and p53 suppressed p53 oligomer formation in unstressed cells and in cells exposed to DNA damage. Furthermore, purified RBEL1A blocked the oligomerization of recombinant p53 corresponding to residues 315-360 in vitro. RBEL1A also significantly reduced the oligomerization of the exogenously expressed C-terminal region (residues 301-393) of p53 in cells. Overexpression of RBEL1A (as seen in human tumors), also suppressed oligomerization by endogenous p53. Our results also showed that GTPase domain of RBEL1A at residues 1-235 was sufficient to block p53 oligomerization. Furthermore, silencing of endogenous RBEL1A significantly enhanced the formation of p53 oligomeric complex following ultraviolet radiation-mediated DNA damage and RBEL1A knockdown also enhanced expression of p53 target genes. Taken together, our studies provide important new molecular insights into the regulation of p53 and the oncogenic role of RBEL1A in the context to human malignancy.

**Implications:**

Elevated RBEL1A expression in human tumors could negatively regulate p53 by inhibiting its tetramerization.

## INTRODUCTION

The p53 tumor suppressor is activated in response to various stresses induced by physical and chemical mutagens, and thereby safeguards genome integrity by preventing the acquisition of genomic alterations. p53 predominantly functions as a transcription factor and to date, more than 60 p53 target genes have been identified that are implicated in regulation of (i) cell cycle arrest, (ii) DNA repair, iii) apoptosis/senescence [1, 2]. Clearly, p53 is an important molecule and its inactivation is believed to be a critical step in tumorigenesis.

In unstressed cells, p53 exists as monomers that are constitutively ubiquitinated by various E3 ligases, such as MDM2, and rapidly degraded [3-5]. In response to cellular stresses including DNA damage, the p53 stability is enhanced as the p53 monomers oligomerize to form dimers, and subsequently two dimers oligomerize to form tetramers. It has recently been reported that in unstressed cells, p53 appears to exist in a mixture of various oligomeric states but following DNA damage, it rapidly assumes a tetrameric conformation [6]. The structure of the tetramerization domain (TD) of p53 has been resolved by different groups using X-ray crystallography [7-8] and NMR [9]. The p53 TD is a hairpin-like structure, consisting of a β-strand (amino acid residues 326-333), an α-helix (amino acid residues 335-356) and a glycine (amino acid residue 334) located at the tip of the hairpin [7]. The computational models suggest that the β-strand from each TD forms an anti-parallel dimer and the α-helices from each dimer oligomerize to form a p53 tetramer giving rise to a tetrameric-helical bundle [10]. It is thought that a p53 tetramer is formed by dimerization of two dimers and this step is crucial for p53 activation as well as DNA-binding to initiate transactivation of target genes [10]. It has also been shown that although p53 dimers are capable of binding to DNA, formation of tetramers can enhance p53 DNA binding affinity by more than 50 folds [11]. The p53 tetramer is believed to behave as a pair of DNA clamps that, with concurrent interactions of both dimers, allows dramatic stabilization of p53-DNA binding [11]. Thus, p53 tetramerization is essential to its function as a transcription factor.

Given the importance of tetramerization, it is not surprising that some cancer cells have acquired various strategies to escape from p53-mediated cellular control by inhibiting p53 tetramerization. In this context, a number of proteins have been documented inhibiting p53 oligomerization. For example, it has been demonstrated that S100B protein specifically interacts with the TD of p53 monomers but rarely with the p53 tetramers, thus, shifting the equilibrium in favor of monomeric conformation with an inhibitory effect on p53 transactivation function [12-13]. It has also been shown that Apoptosis Repressor with Caspase recruitment domain (ARC) protein also interacts with p53 and interferes with p53 tetramerization [14]. These studies demonstrate that inhibition of p53 oligomerization is one of the important mechanisms in negative regulation of p53's function.

We have previously identified a novel Ras-superfamily protein, namely RBEL1A [15-16]. In our previous studies, RBEL1A has been shown to be overexpressed in multiple human malignancies, including breast and colon tumors [15]. RBEL1A is a glycosylated protein that harbors a Ras/Rab-like GTPase domain at its N-terminus [15]. Furthermore, depletion of RBEL1A causes severe growth suppression in cancer cells [16]. Our subsequent studies have further demonstrated that RBEL1A facilitates MDM2-mediated p53 protein ubiquitination and degradation [17]. Depletion of RBEL1A prolongs p53 half-life associated with increased p53 levels; whereas overexpression of RBEL1A reduces p53 levels under unstressed and genotoxic stressed conditions [17]. Our previous studies also have indicated that RBEL1A interacts with oligomeric domain of p53 [17], and thus raises the possibility that such interactions may interfere with the formation of p53 tetramers.

In this study, we sought to examine whether RBEL1A-p53 interactions interfere with p53 tetramerization and transactivation. Our current results indicate that RBEL1A blocks p53 oligomerization in vitro and inside the cells. Our results further indicate that RBEL1A GTPase domain (at residues 1-235) alone is sufficient to block p53 oligomerization. Depletion of RBEL1A in cells also enhanced the formation of p53 oligomeric complex in response to UV-mediated DNA damage. Collectively, our results demonstrate that, in addition to its function in enhancing MDM2-mediated p53 ubiquitination, RBEL1A negatively regulates p53 function by blocking p53 oligomerization.

## RESULTS

### RBEL1A blocks p53 oligomerization

**Figure 1:**
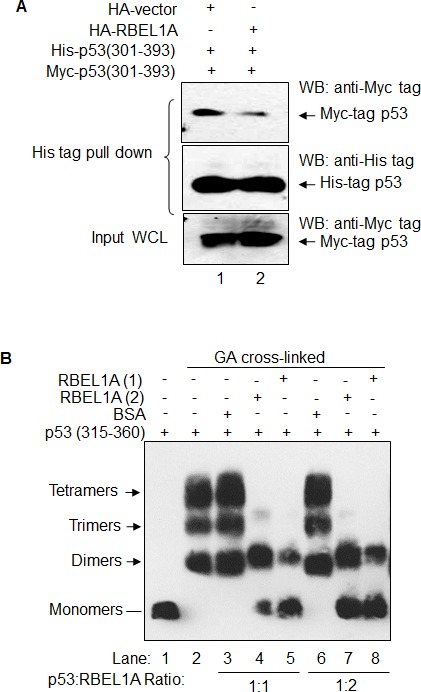
RBEL1A interacts with p53 and blocks p53 (C-terminus) monomer interaction **(A)** RBEL1A inhibits p53 (301-393) monomer interaction in cells. HEK293T cells were co-transfected with vectors carrying His-tagged p53 (301-393) and Myc-tagged p53 (301-393) together with HA-tagged RBEL1A expression vector or HA-tag empty vector. Twenty four hours after transfection, cells were lysed and His-tag protein pull-down assays were performed as described in Materials and Methods. The precipitants were analyzed by Western blotting using anti-myc tag antibodies to determine the interaction between His-tagged and Myc-tagged p53 (upper panel) in the absence (lane 1) or presence (lane 2) of RBEL1A. Middle panel shows the levels of His-tagged p53 (301-393) recovered from the His-tag protein pull-down. Bottom panel shows the expression levels of Myc-tagged p53 (301-393) in the whole cell lysates (WCL, 8%) used for His-tag pull-down assays. **(B)** RBEL1A prevents recombinant p53 (315-360) oligomerization *in vitro*. *In vitro* p53 oligomerization assays were performed as described in Materials and Methods using the purified recombinant p53 (315-360) incubated either with recombinant RBEL1A or BSA prior to crosslink by glutaraldehyde (GA). RBEL1A (1) and RBEL1A (2) were different purifications of RBEL1A protein. RBEL1A protein in purification (1) has larger molecular mass (glycosylated) whereas purification (2) contains RBEL1A with smaller molecular mass (unglycosylated or less glycosylated). The reaction mixtures were separated by SDS-PAGE and the p53 oligomers (as indicated by arrows) were visualized by western blotting using anti-p53 antibody.

In our previous study, we have demonstrated that RBEL1A interacts with p53; the endogenous p53 and RBEL1A associate with each other in cells and the purified recombinant p53 and RBEL1A proteins form complex *in vitro* [17]. Furthermore, RBEL1A interacts with p53 at the C-terminal region of p53 [17], which is critical for p53 oligomerization [7]. For the current studies, we tried to examine whether RBEL1A could interfere with p53 oligomerization and function via its association with p53's C-terminus. To that end, we first generated two different vectors each expressing the C-terminus of p53 corresponding to residues 301-393, one of which was tagged with the His-tag (His-tag p53^−^301-393) and the other fused with the Myc-tag (Myc-tag p53-301-393). Several studies have shown that p53 tetramerization domain alone can spontaneously form tetramers [7, 28]. Thus, we first sought to determine whether RBEL1A interferes with the interactions among the C-termini of p53 i.e. His-tagged and Myc-tagged p53-301-393. As shown in Figure 1A, the His-tagged p53-301-393 was capable of pulling down the Myc-tagged p53-301-393 (Figure 1A, lane 1 upper panel), indicating that the C-terminal region of p53 fused with different tags does indeed exhibit interactions. Figure 1A also shows that, in the presence of RBEL1A, the interaction between the His- and Myc-tagged p53-301-393 was significantly reduced (lane 2, upper panel). These results suggest that RBEL1A disrupts the interactions among the C-terminal monomers of p53.

To further investigate the negative effect of RBEL1A on p53 oligomerization, we performed *in vitro* interaction assays using the purified recombinant proteins corresponding to the (i) C-terminal variant of p53 containing residues 315-360 (p53-Oligo-D) [18] and (ii) purified full-length RBEL1A. The purified p53-Oligo-D was incubated with the recombinant RBEL1A protein (at 1:1 or 1:2 molar ratios) prior to treatment with chemical cross-linker glutaraldehyde (GA). GA has been used in several previous studies of p53 oligomerization [19, 29]. As a negative control, the bovine serum albumin (BSA) was also used instead of the purified RBEL1A. The reaction products were then analyzed by anti-p53 immunoblotting to observe the oligomerization of p53. As shown in Figure 1B, the p53-Oligo-D existed as monomers in the absence of GA (lane 1) but formed typical oligomers corresponding to dimers, trimers and tetramers in the presence of GA (lane 2). However, pre-incubation of p53-Oligo-D fragments with purified RBEL1A (1:1 ratio) but not with BSA (1:1 ratio) clearly inhibited oligomerization by p53-Oligo-D (Figure 1C, compared lane 3 with lanes 4 and 5). Furthermore, increasing the amount of RBEL1A to p53 at 2:1 molar ratio of did not further enhance the effect of RBEL1A on p53-Oligo-D oligomerization (lanes 7 & 8) indicating that RBEL1A:p53 at 1:1 ratio was sufficient to inhibit p53 oligomerization.

**Figure 2:**
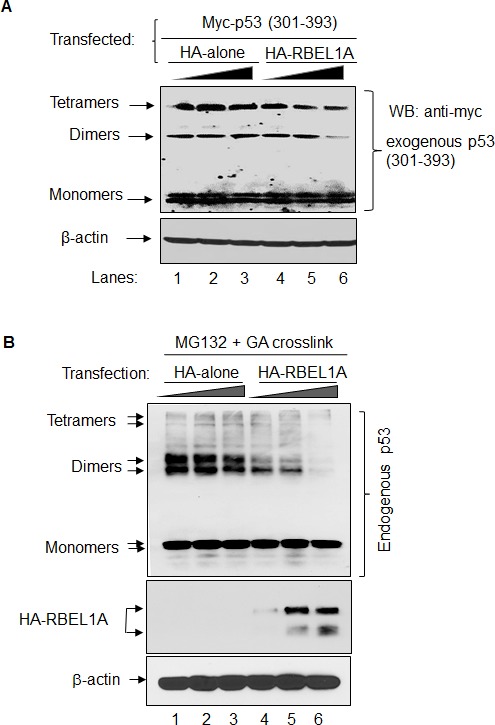
RBEL1A disrupts endogenous and exogenous p53 to form oligomers in cells **(A)** Increased levels of RBEL1A block the formation of oligomeric complexes of the C-terminus of p53 (301-393) in cells. HEK293T cells were transfected with vectors carrying Myc-tagged p53 (301-393) together with increased amount HA-tagged RBEL1A expression vector or HA-tag empty vector. p53 oligomerization was detected by western blot analysis using anti-myc tag antibody. **(B)** MCF12A cells were transfected with either increasing amounts (4, 8, 12 μg) of RBEL1A expression vector or the control empty vector. Approximately 24 hours after transfection, cells were treated with proteasome inhibitor MG132 for 3-5 hour prior to harvesting. Glutaraldehyde (GA, 0.005%) was used to crosslink the cell lysates and incubated for 10 minutes prior to adding the protein loading buffer (with 5% of 2-mercaptoethanol, no boiling) to stop crosslink reaction. The p53 monomers and oligomers were detected on the same membrane. The expression of HA-RBEL1A was also shown and β-actin was shown as the loading control.

Next, we investigated the effect of RBEL1A on p53 oligomerization inside the cells and for that purpose, HEK293T cells were transiently transfected with Myc-tagged C-terminus-p53 (p53-301-393) expression construct along with increasing amounts of RBEL1A expression vector or control empty vector. As shown in Figure 2A, increasing amounts of RBEL1A vector, but not that of the control vector, led to a gradual decrease in the levels of p53 dimers and tetramers (compared with lanes 4-6 with lanes 1-3). We next determined whether RBEL1A affects oligomerization of endogenous p53 inside the cells. In order to observe the effect of RBEL1A on p53 oligomerization, cells were introduced with increasing amounts of RBEL1A expression vector or control empty vector and then were treated with proteasome inhibitor MG132 to prevent RBEL1A-triggered p53 degradation [17]. As shown in the Figure 2B, increasing amount of RBEL1A also led to a gradual decrease in the levels of endogenous p53 dimmers and tetramers. Thus, our results involving *in vitro* as well as in-cell (intracellular) assays indicate that RBEL1A interferes with p53 oligomerization and that it does so via its direct interactions with the p53 oligomerization domain.

### RBEL1A GTPase domain (residues 1-235) alone is sufficient to block p53 to form oligomers

**Figure 3:**
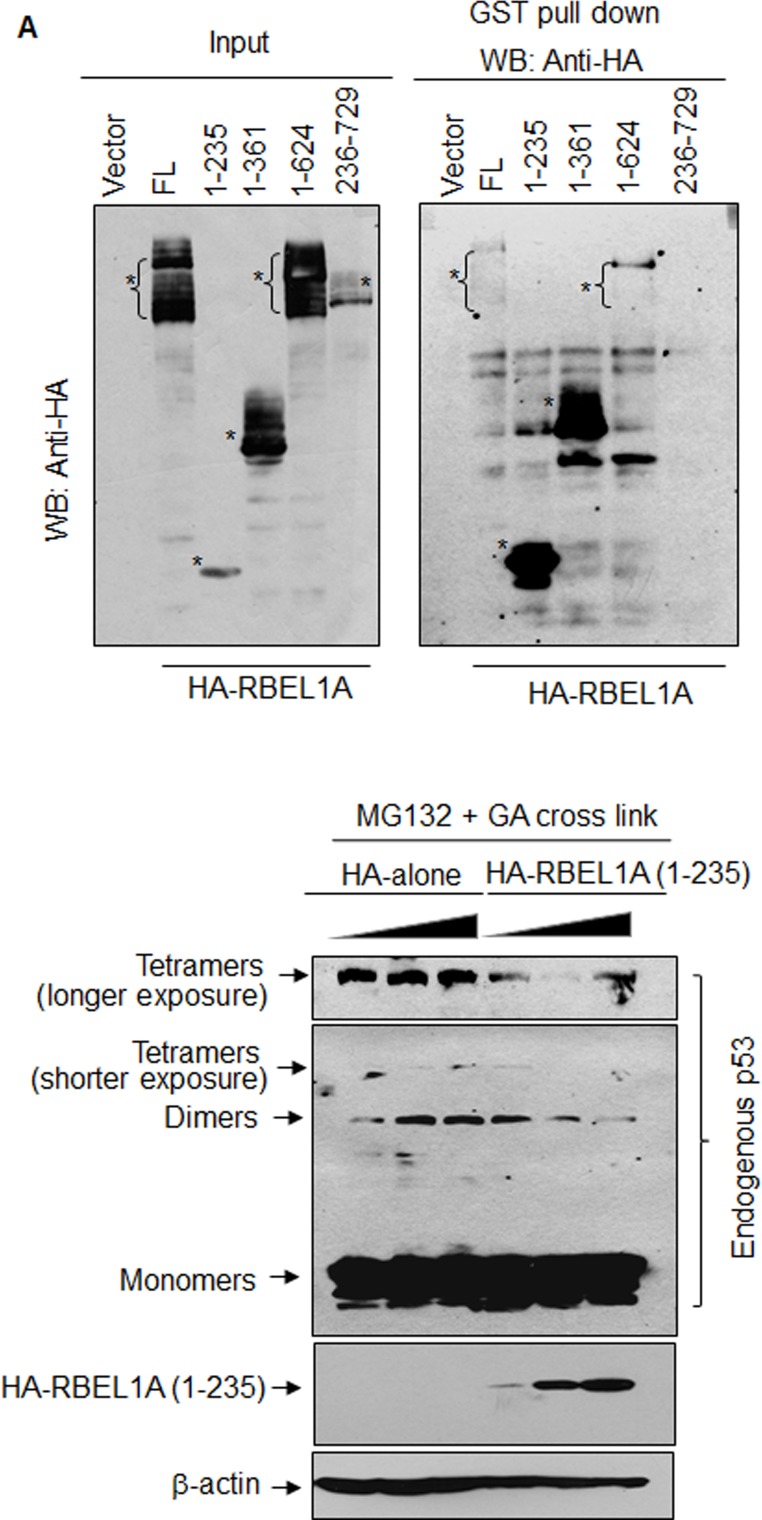
RBEL1A GTPase domain (residues 1-235) alone is sufficient to block p53 oligomer formation **(A)** RBEL1A interacts with p53 via its N-terminus (a.a.1-235). Left panel: Exogenously expressed HA-tagged RBEL1A (full-length and deletion variants) in input lysates of HEK293T cells detected by western blotting using anti-HA antibodies. Asterisk (*) indicates the correctly sized RBEL1A proteins (full-length or deletion variants). Right panel: purified full-length GST-p53 was incubated with cell lysates expressing the HA-tagged RBEL1A (full-length or deletion variants). GST-pull down assays were performed as previously described (ref. 17) and the pull-down products were analyzed for interaction of RBEL1A variants by western blotting using anti-HA antibodies. Numbers refer the RBEL1A residues comprising in the deletion variants. **(B)** MCF7 cells were transfected with increasing amounts (4, 8, 12 μg) of RBEL1A (1-235) expression vector or the control empty vector. Approximately 24 hours after transfection, cells were treated with proteasome inhibitor MG132 for 3-5 hour prior to harvesting. Glutaraldehyde (GA, 0.005%) was added to the protein lysates and incubated for 10 minutes prior to adding the protein loading buffer (with 5% of 2-mercaptoethanol, no boiling) to stop crosslink reaction. The p53 monomers and oligomers were detected on the same membrane and the upper panel shows longer exposure of the image of p53 tetramers. The expression of HA-RBEL1A (1-235) was also shown and β-actin was shown as the loading control.

Next, we sought to determine the minimal region of RBEL1A that was required to inhibit p53 oligomerization. We have previously demonstrated that RBEL1A interacts with p53 via its N-terminal GTPase domain (residues 1-235) that shares high degree of homology with other RAS superfamily GTPAses [17] and results in Figure 3A reconfirm this finding. In light of these results, we sought to determine whether the N-terminal region of RBEL1A (GTPase domain) is sufficient to interfere with p53 oligomer formation. In HEK293T cells, we introduced the HA-only or HA-RBEL1A (residues 1-235) expression constructs and western blotting was performed to examine the p53 oligomer formation in the presence or absence of exogenous RBEL1A. Figure 3B shows that increased amounts of HA-tagged RBEL1A, but not the HA-tag-only, led to a gradual decrease in the levels of p53 dimers and tetramers. Thus, these results demonstrate that the GTPase domain (a.a. 1-235) of RBEL1A alone is sufficient to inhibit oligomerization of p53.

### Depletion of RBEL1A increases p53 oligomerization and transcriptional activity

**Figure 4:**
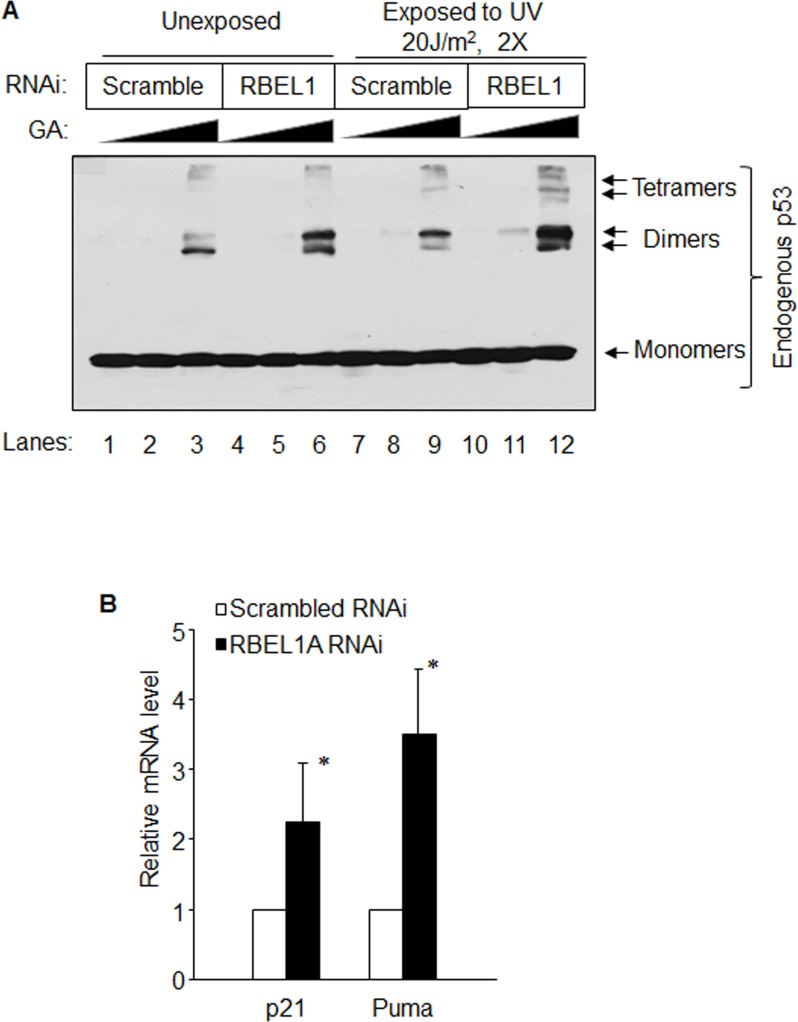
**(A)** Depletion of RBEL1A increases oligomerization of endogenous p53 under unstressed and genotoxic stressed conditions. MCF7 cells were infected with lentiviral RBEL1A shRNA or scrambled shRNA as described in Materials and Methods as we previously described [16, 17]. The lentiviral RBEL1A shRNA or scrambled shRNA were used in our previous studies [16, 17]. Ten days after infection, cells were either unexposed or exposed to UV radiation (20J/m^2^, 2 times instantly). About 4-hours post UV exposure; cells were treated with proteasome inhibitor MG132 for 3 hour prior to harvesting. Increased concentrations (0%, 0.001% and 0.005%) of glutaraldehyde (GA, for protein crosslinking) were then used in cell lysates of UV exposed or unexposed cells. The p53 monomers and oligomers were detected on the same membrane using anti-p53 antibody (FL393). **(B)** Depletion of RBEL1A increased p53 target gene (p21, PUMA) mRNA expression. Results of real-time qPCR of p21 and Puma transcripts in scramble shRNAi and RBEL1A shRNAi expressing cells. Real-time qPCR assays were performed as described in our previous studies [17] and Materials and Methods. The data presented are based on experimental results collected from three-independent experiments performed in three individual triplicates for each sample. (*) indicate p-value <0.01 respectively as determined by t-test.

We also investigated the impact of RBEL1A knockdown (KD) on p53 oligomerization and transactivation of its target genes. MCF7 human breast cancer cells that harbor wild-type p53 were used in which RBEL1A was knocked down via shRNA approach. The cells harboring RBEL1A shRNA or scramble shRNA were either not treated or exposed to UV irradiation (as described in Materials and Methods). For all cell lysates, glutaraldehyde (GA) was used to cross-link p53 proteins prior to the protein denaturation in the loading buffer and oligomer formation of p53 was subsequently determined by western blotting. As shown in Figure 4A, under unstressed condition, the levels of p53 dimer, but not tetramer, were modestly higher in the cells harboring RBEL1A shRNA as compared with those containing the scramble shRNA (compare lane 6 to lane 3). Interestingly, after UV exposure, the levels of p53 dimer and tetramer in the RBEL1A shRNA expressing cells were significantly higher than those detected in the scramble shRNA carrying cells (compare with lane 12 to lane 9). These results indicate that RBEL1A appears to block p53 oligomer formation, especially in cells exposed to DNA damage and silencing of RBEL1A favors p53 oligomer formation. In parallel, we also noted that RBEL1A knockdown increased mRNA expression of p53 target genes including p21 and PUMA (Figure 4B). Taken together, these results indicate that RBEL1A negatively regulates p53 oligomerization and that is coupled with inhibition of p53 transcriptional activity.

## DISCUSSION

**Figure 5:**
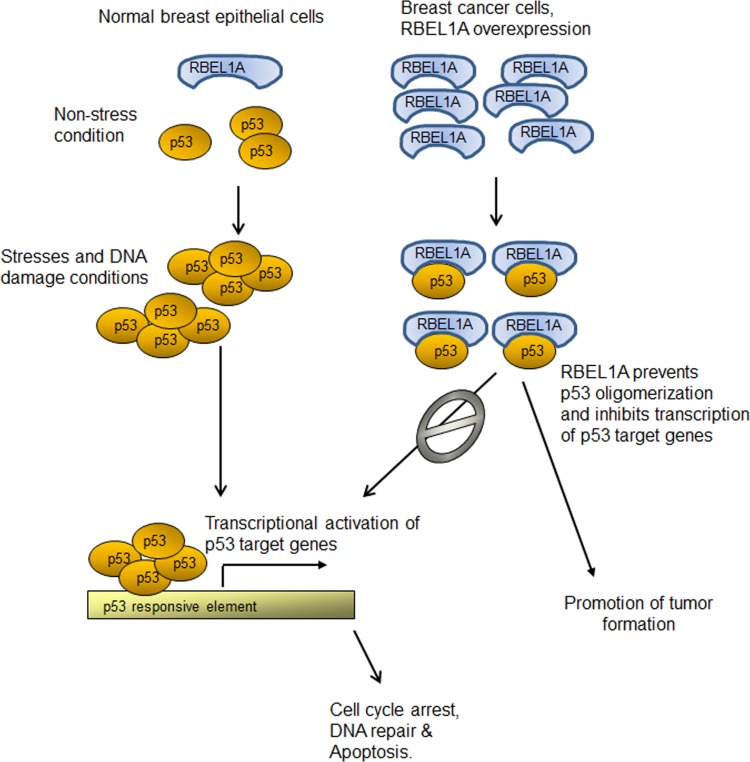
Schematic illustration of the proposed model by which RBEL1A negatively regulates p53 and promotes tumor formation In normal unstressed cells, p53 is expressed at low levels and exists predominantly as monomers and dimers. RBEL1A expression level is also low in the normal cells (15). Following DNA damage, p53 is phosphorylated, stabilized and forms tetramers to transactivate its target genes that induce cell cycle arrest, DNA repair and apoptosis. In cancer cells, RBEL1A is significantly overexpressed [15, 30]. RBEL1A directly interacts with p53 and prevents p53 to form dimers and tetramers. Therefore, RBEL1A blocks the transactivational function of p53 in inducing cell cycle arrest and apoptosis following DNA damage which favors tumor formation.

Previous studies have shown that oligomerization is one of the crucial steps for p53 activation and important for its tumor suppressor function [20-21]. Under unstressed conditions, p53 is believed to predominantly exist as latent monomers, however, when cells encounter intracellular or extracellular stresses, the monomers rapidly oligomerize to form dimmers, and then tetramers. Tetramerized p53 then binds to and transactivates promoters of various target genes that are linked to different functions such as cell cycle arrest, cell death, cellular senescence and DNA repair [2]. In light of the importance of p53 oligomerization, proteins that interfere with p53 oligomer formation are expected to profoundly affect p53 function. Several proteins have been shown to interact with the tetramerization domain of p53 and affect p53 oligomerization. For example, S100A and S100B, members of the S100 protein family, have been shown to interact with the tetramerization domain of p53, and such interaction leads to the blockade of p53 oligomerization [13-14, 22]. The expression of S100A4 and S100B was found to be elevated in a number of human malignancies [23-24]. Studies have also shown that Apoptosis Repressor with Caspase recruitment domain (ARC) protein also regulates p53 tetramerization by its direct binding with p53 tetramerization domain [14]. Knockdown of ARC in breast cancer cells led to nuclear accumulation as well as increased tetramerization of p53 coupled with activation of p53 target genes [14]. Interestingly, elevated ARC expression was also noted in human cancer cells [25]. Likewise, our results suggest that RBEL1A may function in a similar fashion to regulate p53 tetramerization. Our previous studies demonstrated that RBEL1A interacts with p53 at p53's tetrameric domain [17] and our current studies show that the interactions between p53-RBEL1A inhibit p53 oligomerization (Figure 1-3). Our results suggest that RBEL1A:p53 1:1 ratio is sufficient to prevent p53 to form oligomers (Figure 1C). Importantly, our results also demonstrate that following DNA damage, depletion of RBEL1A led to increased p53 oligomerization (Figure 4A) and RBEL1A knockdown also increases p53 target gene expression (Figure 4B). In context to these findings, our previous studies have shown that RBEL1A is overexpressed in multiple human malignancies including breast cancer and colon cancer [15]. More recently a study by Li et al [30] have also shown that RBEL1A expression is significantly elevated in a large portion of primary breast cancers and high levels of RBEL1A expression correlates with poor survival in breast cancer patients [30]. Based on our collective findings from current and previous studies, we propose a possible model via which RBEL1A negatively regulates p53. As is shown in Fig. 5, in normal unstressed cells, p53 is expressed at very low levels and exists predominantly as monomers and dimers. The expression levels of RBEL1A are also low in the normal cells (15, 30). In response to DNA damage, p53 is phosphorylated, stabilized and forms tetramers to transactivate p53 target genes that induce cell cycle arrest, DNA repair and apoptosis. In cancer cells, RBEL1A expression levels are significantly elevated [15, 30]. Abundant RBEL1A are available for interacting with p53, preventing p53 dimer and tetramer formations and sequestering p53 in the monomeric state. Such action of RBEL1A blocks the transactivational function of p53 in inducing cell cycle arrest and apoptosis following DNA damage which favors tumor formation.

Interestingly, our studies also demonstrate that RBEL1A GTPase domain alone (residues 1-235) is sufficient to interfere with p53 oligomer formation (Figure 3B). These findings would suggest that the physical interaction between RBEL1A and p53, rather the GTP-binding potential or the GTPase activity of RBEL1A, appears to be critical for RBEL1A to obstruct p53 tetramer formation. Presumably, RBEL1A-p53 interaction prevents p53 monomers to form the oligomeric complex because the oligomeric region is engaged by RBEL1A. Our previous studies had demonstrated that the GTP-binding defective mutants of RBEL1A were still capable of interacting with p53 [17]. Based on these findings, it is possible that the GTP-binding activity of RBEL1A is not required for its interaction with p53. Future studies are needed to further dissect the molecular details of RBEL1A-mediated regulation of p53 oligomerization. Our current studies thus provide additional new information about the mechanism by which p53 function is regulated and also the molecular insights into the oncogenic role of RBEL1A in the context to human malignancy.

## MATERIALS AND METHODS

### Antibodies and reagents

Antibodies specific to p53 (DO-1 and FL393), His-tag and Myc-tag were purchased from Santa Cruz Biotechnology (Santa Cruz, CA). Antibody specific to HA-tag (HA.11) was from Convance (Berkeley, CA) and those for α-tubulin and β-actin were from Sigma (St. Louis, MO). Polyclonal RBEL1 antibody was generated in our laboratory [15-16]. Proteasome inhibitor MG132 was purchased from Sigma (St. Louis, MO), glutaraldehyde (GA) and bovine serum albumin (BSA) were from Fisher Scientific Inc (Pittsburg, PA).

### Expression constructs and RBEL1A RNAi

Construction of HA-S-tagged full-length RBEL1A and its deletion variants 1-235 have previously been reported [17]. Carboxyl-terminal His-tagged and Myc-tagged pSRα-p53 (301-393) were generated by inserting the PCR-amplified cDNA fragment into pSRα vector. All expression vectors were verified by DNA sequencing. The RBEL1A RNAi and scramble RNAi were used in our previous studies [16, 17].

### Cell culture, transfection and RBEL1A knockdown

RKO, MCF7 and HEK293T were cultured in Dulbecco's modified Eagle's medium (DMEM) supplemented with 10% fetal bovine serum (Gemini Bio-Products Inc., West Sacramento, CA), 10 I.U/ml Penicillin-Streptomycin (Mediatech, Inc, Manassas, VA) and 200 μM L-glutamine (Mediatech, Inc). Human non-tumorigenic breast MCF-12A cells (kindly provided by Dr. G. Wayne Zhou, Marine Biological Lab, Woods Hole, MA) were maintained in DMEM supplemented with 10% FBS and 10 μg/ml insulin, 20 ng/ml hEGF, 100 ng/ml cholera toxin, 500 ng/ml hydrocortisone, 10 I.U/ml Penicillin-Streptomycin and 200 μM L-glutamine (Mediatech, Inc). For RBEL1A overexpression experiments, indicated cells were transfected using lipofectamine 2000 (Invitrogen Life Science, Grand Island, NY) according to manufacturer's procedure. For RBEL1A knockdown experiments, MCF7 cells were infected with RBEL1A specific shRNA (RBE L61, GAAGAATGACTCGGACCTCTTCTCGAGAAGA GGTCCGAGTCATTCTTC) or scrambled shRNA lenti-viral particles (MOI=1) overnight [16]. The infected cells were then selected by puromycin (2 ng/ml); and RBEL1A-knockdown and scrambled shRNA cells were used and analyzed 10 days after infection.

### Immunoblotting and immunoprecipitation analyses

Immunoblotting and immunoprecipitation were performed as we have previously described (17, 26).

### His-tag pull down assay

Myc-tagged and His-tagged p53 (residues 301-393) were co-transfected with pSRα-RBEL1A or HA-alone vectors in HEK293T cells. Twenty-four hours later, cells were harvested, lysed and ~3 mg of total protein were then mixed with 60 μl of Ni-NTA His•Bind Resin and incubated and rocked at 4°C for 5 hours. Protein-binding resins were washed extensively and eluted by loading buffer containing 5% β-mecaptoethanol. Proteins from the elutes were separated by SDS-PAGE gel and western blots were performed using anti-Myc or anti-His antibodies.

### In vitro p53 oligomerization assay

Purified recombinant polypeptide, harboring the p53 tetramerization domain (TD) (residues 315-360) [18], was incubated with either the purified recombinant RBEL1A protein or bovine serum albumin for 1 hour at 4°C and then cross-linked with glutaraldehyde (GA) for 5 minutes at 4°C. The mixture was resolved by SDS-PAGE gel and immunoblotting was performed using polyclonal anti-p53 antibody (FL-393, Santa Cruz).

### In-cell p53 oligomerization assay

The RBEL1A full-length and deletion-mutant overexpressing cells or RBEL1A knockdown cells with or without exposure to ultraviolet (UV) radiation (20 J/m^2^, 2 times) were treated with proteasome inhibitor MG132 (10 μM) for 5 hours. Cells were then lysed and the soluble cell lysates were extracted. About equal amounts of protein (300-400 μg) from each cell lysate were cross-linked with glutaraldehyde (GA, a final concentration of 0.005%) for 5 minutes and subsequently resolved by SDS gel electrophoresis, and immunoblottings were performed using anti-p53 (FL393) antibody.

### Quantitative real-time PCR (qPCR)

qPCR assays were performed as previous described [17]. The primers used as follows: p21 forward primer: 5′-CAGACCAGCATGACAGATTTC-3′ and p21 reverse primer 5′-TTAGGGCTTCCTCTTGGAGA-3. Puma forward primer: 5′-AGAGGGAGGAGTCTGGGAGTG-3′ and Puma reverse primer: 5′-GCAGCGCATATACAGT ATCTTACAGG-3′. β-actin forward primer: 5′-GCTCGTCGTCGACAACGGCTC-3′ and β-actin reverse primer: 5′-CAAACATGATCTGGGTCATCTTCTC-3′ [17]. Each sample was analyzed in triplicate and repeated at least by three independent experiments. β-actin was used as internal control and normalization. Data analysis used comparative ∆∆Ct method as previous described [17].
